# Electric trains for reduction of fuel consumption and emissions from the freight transport in Jordan

**DOI:** 10.1371/journal.pone.0323121

**Published:** 2025-05-09

**Authors:** Charles Sassine

**Affiliations:** 1 Faculty of Life Sciences, Humboldt University of Berlin, Berlin, Germany; 2 Department of Transportation, Dar, a Sidara Company, Verdun Street Beirut, Lebanon; Guangzhou Institute of Geography, Guangdong Academy of Sciences, CHINA

## Abstract

Transport modes (trucks and diesel trains) used to move phosphate on the corridor connecting Shidiya mine and Aqaba port in Jordan are causing pollutant emissions exceeding global average values. The study proposed the use of electric trains and compared their fuel consumption (FC) and emissions to those of existing transport modes in an attempt to regulate FC and reduce emissions from freight in Jordan. For a more comprehensive view of the three transport modes, FC and emissions were evaluated in various operating conditions in relation to varying speeds, loads, road gradients, and curvatures. Results showed that the three vehicle types were greatly impacted by increasing payloads and speeds. On the other hand, diesel trains were less affected by the road’s curvature and more by its gradient. Electric trains can significantly reduce FC and CO_2_ emissions at 100% payload and an average speed of 60 km/h compared to trucks and diesel trains. Also, the use of electric trains would maintain CO and N_2_O emissions at acceptable levels. Regression analysis demonstrated significant negative correlations (R^2^ > 0.9) between FC and pollutant emissions with increasing payloads for the three vehicles. However, positive correlations were obtained with increasing speeds for diesel (R^2^ = 0.91) and electric trains (R^2^ = 0.77). The study offers insights into Jordan’s future strategies for reducing pollution in the transport sector. Based on the findings of the study, drivers may preferably use 100% payload and maintain an average speed of 60 km/h on the selected corridor. Besides, the use of renewable energy sources could potentially further reduce carbon emissions from electric rails.

## Introduction

One of the primary sources of emissions contributing to urban pollution is the transport sector [1]. In 2021, the global transportation sector accounted for 37% of total carbon dioxide (CO_2_) emissions, and in 2022, shipping was the second most carbon-intensive subsector, after road vehicles, contributing 11% of global CO_2_ emissions [2]. In addition to carbon dioxide, the operation of roads, trains, and other modes of transport generates significant amounts of other pollutants, such as particulate matter (PM10 and PM2.5), nitrogen oxides (NOx), carbon monoxide (CO), and sulfur oxides (SOx) [3]. Road freight transport is the main source of emissions [4]. It accounts for more than 70% of total emissions [5] and contributes significantly to CO_2_ emissions because it relies on fossil fuels [6,7]. As a result, it poses a severe risk to the quality of the air in cities [8,9]. Nevertheless, the amount of fuel consumed by various transportation modes has a substantial impact on their CO_2_ emissions [10]. Railway transportation uses less energy and produces less pollution compared to road transportation [11,12]. Trains have a high capacity for carrying freight, resulting in relatively higher energy efficiency per unit of cargo transported [13]. Blevins and Gibson [14] demonstrated that for freight transport, railroads could save 65–70% on fuel as compared to trucks. Also, shifting freight from truck to rail in the upper Midwestern United States reduced CO_2_ emissions by 31% [15]. Further, a case study conducted by Barth and Tardi [16] for the California Interstate 40 corridor, found that rail freight transport produces lower CO emissions than trucks. Although regarded as a more environmentally friendly option than trucks, train systems emit pollutants into the atmosphere which are harmful for air quality [17,18]. On the other hand, transport-related emissions can be further reduced by electrifying railways because electric trains directly eliminate emissions from the combustion of fossil fuels [19,20]. According to Shirres et al. [21], they are twice as powerful as diesel locomotives and the only net-zero carbon option for freight. Nonetheless, the energy source used to produce electricity is important [22]. For instance, using renewable energy sources like wind, solar, or hydropower, or fuels like hydrogen and biodiesel to power trains, reduces emissions by removing direct emissions from trains and maximizing energy efficiency [23].

Electrification of railway transportation is thus a promising alternative to road for reducing pollution [3,20,24]. According to a research in China [25], electrifying railways and using a mix of renewable energy sources could reduce carbon emissions by 65.4%.

In Jordan, the transportation sector accounts for more than 8% of GDP [26] and contributes to 4,706 Gg CO2e [27]. It is expected to grow at a 5–6% annual rate until 2030 [26]. The Jordanian government uses a century-old 294 km narrow-gauge railway line to transport phosphate from the Shidiya Mine (in Maan Governate) to Aqaba Port (in Southern Jordan). Other rail sections, totaling about 210 km, are currently inactive [28]. Trucks are also used for phosphate transport, but the use of low-quality fossil fuels for transport vehicles aggravates air pollution by causing pollutants, such as NOx, CO_2_, and PM2.5, to exceed global average values [29]. According to their assessments of Jordan’s railway system, Al-Asmara [30] and Rjoub and Shawabkeh [31] recommended that existing railways be developed and reactivated.

Besides, enhancing energy efficiency and environmental safety in the transport industry is one of the emerging priorities in the global economy [32,33]. Factors affecting fuel consumption and emissions vary based on vehicle type and operating conditions, as discussed in previous research studies. For instance, vehicle weight, technical speed, load factor, and vehicle age have been considered by Goryaev et al. [34] as main factors affecting trucks fuel consumption. Besides, Madre et al. [35] and Rizet et al. [36] reported that both speed and payload affected the fuel consumption of heavy-duty vehicles. Turkensteen [37] found that speed is the most important factor affecting fuel consumption of trucks. According to McKinnon [38], increasing the vehicle load may improve energy efficiency, reduce road transport costs, and reduce CO_2_ emissions from freight transportation. Increasing track gradient could also affect the fuel consumption of trucks [39]. In another study, Fullerton and Dick [40] reported that the train load had the greatest influence on fuel efficiency compared with speed and gradient. Energy consumption and emissions of a vehicle are also highly influenced by road curvature and driving behavior [41, 42, 43].

This study considered the existing road and railway options used for phosphate transport on the Shidiya mine-Aqaba port corridor, comparing energy consumption and emissions to proposed electric trains in an attempt to evaluate the potential implementation of the latter for freight transport in Jordan. The study also evaluated the effect of several factors, such as payload, speed, road gradient, and curvature on energy consumption and emissions to identify the factors that have the highest influence relatively to each transport mode. While most of early studies in this field have focused on the effect of these factors on a single transport mode, this study compared their effects among three types of vehicles; trucks, diesel trains, and electric trains in different operating conditions aiming to inform government strategies for regulation of fuel consumption and emissions reduction from the freight transport sector in Jordan.

## Materials and methods

### Study area and approach

The corridor considered in this study is situated between the Shidiya mine in the Maan Governorate and the Aqaba port in Jordan. The corridor includes road and rail lines crossing a variety of topographical features, ranging from mountains to deserts. The study has considered the years 2020, 2021, and 2022, considering different volumes of phosphate exported from the Shidiya mine.

### Description of transport modes

Based on the annual reports of the Jordan Phosphate Mines Company [44–46], 2.3, 2.9, and 3.4 million tons of phosphate were transported, in total, on the studied corridor during 2020, 2021, and 2022, respectively, over a 170 km stretch. Large trucks (HGV; 3 axles, 10 tires) that can transport 40 tons per trip are utilized for freight transportation. A total of 37,500 vehicles must operate for 300 days a year, or 125 trucks per day, to transport the specified volumes [28]. There are three trains of narrow-gauge 1,050 mm New Zealand DH class GE U10B diesel locomotives on this line. Trains have a 1,650-ton capacity (40 wagons per train). To convey the required volumes, 900 trains must run 300 days a year, or three trains each day with four locomotives each. Since there are no electric locomotives in Jordan, the study suggested the electric version of the New Zealand EF class EF 30042 narrow-gauge locomotive (about 1,067 mm) which has transport characteristics comparable to those of diesel trains (1,650 T capacity, 40 wagons per train). Therefore, three trains (four locomotives each) are required daily, and for 300 days of operation, 900 vehicles are required.

### Calculation of fuel consumption

Considering truck and diesel train options, the annual fuel consumption (FC) for road and rail transport was computed and compared across the indicated stretch, assuming similar phosphate volumes transported for both. Additionally, calculations were made under the assumption that the proposed electrical railway would deliver the total volumes of phosphate indicated for the same three years.

#### • Roads.

Fuel consumption (FC) calculations for roads were based on Thoresen’s [47] model, which uses the NIMPAC (NAASRA) Improved Model for Project Assessment and Costing (1).


Fuel\ Consumption (FC)=BFC (1+EEA+GA+CA+RRA+CA)

Where: *BFC: Basic Fuel Consumption, EEA: Engine Efficiency Adjustment,* GA: Grade Adjustment, CA: Curvature Adjustment, RRA: Road Roughness Adjustment*, CoA: Congestion Adjustment*

The fuel/speed relationship is a polynomial that predicts fuel consumption on a flat, straight road at a constant speed, with no traffic congestion (2).


$$\text{BFC}=\text{k}+{\text{av}}^{2}+\;\frac{\text{b}}{\text{v}}$$ (2)

*Where: Basic Fuel consumption (1/1000 km), k: constant, v: speed, a: speed squared, and b: speed reciprocal.*

Basic fuel consumption for the HGV truck was calculated using the NIMPAC model, resulting in 215 liters per 1,000 km at 60 km/h. The study considered seven speeds increasing from 30 to 90 km/h, at a rate of 10 km/h, with adjustment factors computed to reflect fuel consumption variations. According to BTRE [48], payload significantly affects fuel consumption, even though it is not considered in Thoresen’s model. A strong linear correlation between basic fuel consumption and payload has been reported. Therefore, in this study, six different payload levels (10%, 25%, 50%, 70%, 90%, and 100%) were selected, and fuel consumption was adjusted using correction factors ranging from 0.8 to 1.18, corresponding to 0% and 100% payload values.

The road segments were analysed for gradient values ranging from -6% to 6%. Fuel consumption (FC) was calculated by multiplying each gradient value by its corresponding adjustment factor. Horizontal curves were also assessed and compared to Thoresen’s curvature types to determine adjustment factors. Additionally, congestion adjustment was calculated (3) according to Parajuli [49].


Congestion\ adjustment=MIN (1, VCR)*FCONG

*Where: VCR: Volume to Capacity Ratio, FCONG: Traffic Congestion Adjustments to Fuel Consumption.*

Road roughness was evaluated for each segment and compared to Thoresen’s [47] classification, ranging from very poor to very good, with roughness factors between 0.05 and 0.

#### • Diesel railway (Rail D).

A common method for calculating energy consumption is the equations of motion (4), which represent the train as a point mass based on mechanical principles [50].


$$m\;\times \;\frac{dv}{dt}=F(v,u)-R(v)+T(x)$$

Where: *m: train mass*, *v: train speed*, *F: Tractive force generated at the wheels*, *u: control setting*, *R(v): resistive force acting on the train*, *T: track force resulting from gradient and curvature*, *x: train location.*

The tractive force required to maintain a constant speed ‘v’ is the difference between the resistive force *R(v)* and the track force *T(x)* (5):


 F=R(v)− T(x)

Where: *F: the net force required to keep the train moving at a constant velocity v, R(v): the resistive force acting on the train, which depends on the speed, and T(x): the track force provided by the train’s propulsion system.*

The resistive force is typically modelled as a quadratic function (6) of speed and is commonly referred to as the Davis equation [51]:


$$R(v)=r_{0}+{(r}_{1\;}\times \;v)+{(r}_{2}\;\times \;v^{2})$$

Based on Lukaszewicz [52], r0 = (65 x n) + (0.9 x 10e-5 x m), r1 = -22 + (0.58 x Lt), and r2 = 5 + (0.58 x Lt), where n is the number of axles, m is the mass of the train, and Lt is the length of the train.

The track force was modelled as below (7):


T(x)=G(x)−C(x)

*Where: G: gradient force acting on the train, and C: the curvature resistance: extra force and energy a train needs to overcome when navigating curves.*

Gradient force is positive on declines and is given by (8):


G(x)=m*g* sin (θ(x))

*Where:* Ɵ*: angle of slope of the track, g: acceleration due to gravity (9.8 m/sec*^*2*^*).*

Curvature resistance (penalty) is expressed as a percentage based on the track’s curvature radius. The curvature resistance is 0.04% per degree of curvature for standard gauge tracks [53–54]. Thus, for each degree of curve, an additional 0.04% of the train’s mass contributes to total resistance. As per Parajuli [49], to account for the curvature penalty, the curvature force or centrifugal force is often expressed as (9):


$$C(x)=\frac{6,33\;x\;mass}{r(x)}*R$$

Where:

*-Factor 6.33 is an empirical constant based on typical train mass and curvature characteristics for standard gauge tracks (1435 mm).*

*-R is the curvature ratio that adjusts the formula based on the specific track gauge. The ratio would be 1 for standard gauge track (1,435 m).*

*-r (x) is the radius of the curvature.*

Specific Fuel Consumption (SFC) is a key indicator of an engine’s fuel efficiency, measuring the fuel needed to produce a certain amount of energy, typically in kg/kWh or kg/J [55]. For this case study, the SFC is 0.23 kg/kWh, which converts to 6.4 x 10 ⁻⁸ kg/J. Additionally, the specific gravity of diesel fuel (0.83) helps convert mass-based fuel consumption to volumetric flow rate. With an SFC of 6.4 x 10 ⁻⁸ kg/J and a specific gravity of 0.83, the volumetric fuel consumption is calculated as approximately 7.7 x 10 ⁻⁸ litres/J.

The fuel consumption for the entire trip is related to the distance of the trip, the power required to maintain a constant speed, and the efficiency of the electric traction system (10).


$$\text{FC}=7,7\;{\text{e}}^{-8}\times \text{d }\times \frac{\left[\text{R}(\text{v})-\text{T}(\text{x})\right]}{\text{n}}$$

Where: *FC = Fuel consumed for the entire trip (litres)*, *d = Distance of the trip (in meters)*, *R: Resistive force T: Track force*, *n = Efficiency of the electric traction system (dimensionless)*

#### • Electrical railway (Rail E).

The instantaneous energy consumption of a train was estimated based on Hickman [56] by combining steady-state load, acceleration energy, and energy for gradients. For traffic-related emissions, energy consumption is integrated over the trip length using the average speed. If the steady-state load is modelled by a second-order polynomial, the integrated energy consumption for the route can be calculated as follows (11):


$$\;E^{\prime }=\left(\frac{\left(N_{Stop}+1\right)}{L}\right)\times \frac{\left(V_{\max}^{2}\right)}{2}+\;B_{0\;}+B_{1}.\;V_{a}\;+B_{2}.V_{a}^{2}+\frac{g.dh}{L}$$

Where: *E’: Energy Consumed in KJ/ton.km, Nstop: Number of Stops, L: Length of Alignment in Km, Vmax: Maximum speed in m/s taken as 22.2 m/s (80 km/h), B0, B1, B2: Coefficients, Va: Average speed in m/s, G: Acceleration caused by gravity is 9.81 meters per second squared, Dh: Elevation difference in km.*

Constants for the above equation are provided by Hickman [56] for freight trains in KN/ton for speed in m/s as follows: B0: 24.7, B1: 0, and B2: 84.5 x 10e-3. The results were converted into specific energy consumption in kWh/km (and subsequently to l/km) using the following formula (12):


$$\frac{KJ}{ton.km}=\frac{Kwh}{km}\;\times \;\frac{KJ}{Kwh}\;.\;[ton{]}^{-1}$$

### Calculation of pollutants emissions

#### Road emissions.

Emissions of pollutants were assessed as affected by different speeds, payloads, gradients, and curvatures. Continuous emission rate functions, dependent on the vehicle’s average speed, were derived through statistical curve fitting using data from the Emission Factors for Road Transport workbook [57] specific to HGV trucks (40 tons) (13):


$$\epsilon =\text{K}+\text{av}+{\text{bv}}^{2}+{\text{cv}}^{3}+\frac{\text{d}}{\text{v}}+\frac{\text{e}}{{\text{v}}^{2}}+\frac{\text{f}}{{\text{v}}^{3}}$$ (13)

Where: ε: emission rate in grams per kilometre for an unloaded goods vehicle on a flat road, K: constant, *v: average speed of the vehicle in km per hour (km/h), a-f: coefficients*. *Coefficients relative to each pollutant were derived from Keller [*57*] for heavy goods vehicles (HGV).*

Based on Ahlvik [58], it is recommended to adjust emissions calculations for vehicles on flat roads based on road gradient (14):


  εg=ε*as

Where: ε*g: emission rate in grams per km, adjusted for gradient;* ε: *emission rate in grams per km for an unloaded HGV vehicle, as: gradient correction factor.*

Gradient factors, which depend on road gradient, pollutant type, and vehicle mean speed, can be calculated as a polynomial function for each vehicle category, gradient, and pollutant, as follows (15):


$$as=(A6*V^{6})+(A5*V^{5})+(A4*V^{4})+(A3*V^{3})+(A2*V^{2})+(A1*V)+A0$$ (15)

Where: *V: Average speed in Km per hour (km/h), A0 - A6: Constants specific to each pollutant, vehicle, and gradient category. Coefficients are derived from Hickman [*56*].*

Due to their impact on overall weight, vehicle loads have a major impact on emissions and fuel consumption in heavy-duty vehicles [59]. Correction functions for load have been developed for goods vehicles (16):


εl=εu*F(g,v)

Where: ε*l: emission rate in grams per km under loaded conditions,* ε*u: emission rate in grams per km for an unloaded vehicle, F(g,v): load correction factor; g: gradient in %, v: mean speed of the vehicle.*

Thus, the load correction factor functions are of the form (17):


$$F(g,v)=K+(n*g)+(p*g^{2})+(q*g^{3})+(r*v)+(s*v^{2})+(t*v^{3})+\frac{u}{v}$$ (17)

Where: *v: average speed in km/h, K: constant, n-u: coefficients. Coefficients were derived from* Ntziachristos and Samaras [59] *for heavy goods vehicles (HGV).*

#### Railway emissions.

Emissions from diesel- and electric-powered train locomotives were estimated by multiplying fuel consumption by an energy-specific emission factor (18). Standard factors for emissions in diesel and electrical railway locomotives were based on Hickman [56].


$${\;E}_{i}=F\;\times EF_{i}$$

Given: *Ei: cumulative emission of pollutant “i” within the given time frame, F: overall fuel consumption during the specified time frame, EFi: emission factor specific to fuel.*

### Data analysis

Calculations of fuel consumption and pollutants emissions were computed using Excell Macro. Statistical analysis applied Factorial ANOVA for the estimation of the effects of the different studied factors on averages of fuel consumption and emissions estimated for the three years of study. Also, regression analysis was performed to determine significant correlations between the factors (speed, payload, gradient, and curvature) with fuel consumption and emissions. A 95% confidence level was used for the different tests.

## Results and discussion

### Total fuel consumption and emissions

Results (Table 1) showed that the total fuel consumption (TFC) of Rail D and Rail E was significantly lower compared to that of trucks, by around 40% and 50%, respectively. Comparing Rail D and Rail E to trucks, the total CO_2_ and total N_2_O emissions were lowered by 45.1% and 80.8% and by 47.4% and 60.5%, respectively. Total PM emissions were significantly lower from Rail E than from trucks. The highest VOC and CO emissions were from Rail D and were significantly higher than those from trucks and Rail E.

**Table 1:** Fuel consumption and pollutants’ emissions from different vehicle types.

	TFC (L/km)	TCO_2_ (g/m3)	TCO (g/m^3^)	TVOC (g/m^3^)	TN_2_O (g/m^3^)	TPM (g/m^3^)
**Trucks**	8200624.7a	28276.3a	74.9b	33.0b	378.8a	18.3a
**Rail D**	4927225.3b	15520.8b	110.9a	69.0a	246.4b	17.3a
**Rail E**	4116752.1b	5434.1c	74.7b	30.6b	149.4b	9.5b
***P value***	0.011	0.001	0.065	0.004	0.005	0.022

TFC: total fuel consumption, Rail D: diesel rail, Rail E: electric rail, Means followed by different letters within the same column show a statistically significant difference at *P value* <0.05 according to Duncan’s Multiple Range test

According to Kazemi and Saki [60], rail can significantly reduce air pollution compared to road transport. Similarly to this study findings, Barth et al. [61] and Comer et al. [62] reported a reduction in fuel consumption, CO_2_, and CO emissions from rails compared to truck transport of freight. However, contrarily to our findings, Comer et al. [62] noted an increase in NOx and PM emissions when shifting from trucks to rail. Font et al. [63] added that when a train runs on diesel instead of electric power, CO_2_ emissions are three times greater and N_2_O levels can rise by up to 14 times. Train emissions are caused by engine exhaust and non-exhaust wear processes. Exhaust emissions from diesel combustion produce CO, VOC, N_2_O, and fine particulate matter (PM2.5) [64]. Non-exhaust emissions that are typical of both diesel and electric trains include coarse particulate matter (PM10) that are released by friction, wheel-rail interaction, pantographs, catenaries, or brake wear [65].

In this case study, CO_2_ emissions from Rail E are reduced by around 81 and 65% compared with trucks and diesel trains. In the UK, running electric trains produces roughly 22% less CO_2_ emissions per vehicle-km than running diesel trains [18]. Total CO_2_ levels recorded from Rail E were lower than the Occupational Safety and Health Administration (OSHA) Permissible Exposure Limit (PEL) [66], while those from Rail D and trucks were higher than this limit.

Further, N_2_O levels from Rail E were lower than the airborne PEL set by OSHA [67], while those from Rail D and trucks were higher than this limit. On the other hand, CO emissions from Rail D exceeded the Jordanian ambient air quality standards’ maximum permissible limits for CO [68], whereas CO emissions from Rail E and trucks fell below this threshold. The three vehicle types released more PM than the ATSDR [69]-designated hazardous range and more volatile organic compounds (VOCs) than the Kephalopoulos et al. [70] recommended range for the overall concentrations of VOCs in urban air.

Those findings showcased electric rails as less fuel consuming and more eco-friendly (especially in terms of CO_2_ emissions) option compared to roads and diesel rails. Using the life cycle assessment methodology (LCA), Merchan et al. [71] concluded that electric trains outperform diesel trains in terms of environmental performance in Belgium. Future research may be oriented towards conducting an LCA analysis to compare the environmental impacts of electric trains to those of conventionally used freight transport modes in Jordan.

### Payload, speed, curvature, and gradient effects

Results in Table 2 demonstrated that the factor “payload” had a statistically significant effect (at *P value* <0.05) on the averages of FC and emissions of all pollutants investigated for the three types of vehicles. The factor “speed” had a significant effect on FC and emissions of Rail D and Rail E as well as on the truck’s VOC, N_2_O, and PM emissions. On the other hand, the means of FC and pollutants emitted from Rail D only were significantly impacted by “curvature” and “gradient” factors. Also, ‘gradient’ significantly affected CO_2_ emissions from trucks.

**Table 2:** Effects of payload, speed, curvature, and gradient on fuel consumption and emissions of three vehicle types.

	Significance *(P value*<0.05)					
Payload (*df: 5,12*)	FC	CO_2_	CO	VOC	N_2_O	PM
Trucks	0.000	0.000	0.000	0.000	0.000	0.000
Rail D	0.000	0.000	0.000	0.000	0.000	0.000
Rail E	0.000	0.000	0.000	0.000	0.000	0.000
**Speed** (*df: 6,14*)						
Trucks	1.000	0.173	0.088	0.001	0.021	0.009
Rail D	0.000	0.000	0.000	0.000	0.000	0.000
Rail E	0.001	0.001	0.001	0.001	0.001	0.001
**Curvature** (*df: 6,14*)						
Trucks	0.960	1.000	1.000	1.000	1.000	1.000
Rail D	0.004	0.004	0.004	0.004	0.004	0.004
Rail E	1.000	1.000	1.000	1.000	1.000	1.000
**Gradient** (*df: 6,14*)						
Trucks	0.859	0.029	0.911	0.818	0.310	0.332
Rail D	0.000	0.000	0.000	0.000	0.000	0.000
Rail E	1.000	1.000	1.000	1.000	1.000	1.000

FC: fuel consumption, Rail D: diesel rail, Rail E: electric rail

### Effect of payload

When the effect of “payload” was compared between the three vehicle types (Table 3), FC recorded values that were comparable for payloads of less than 50%. However, at 75% payload, Rail E’s FC was significantly lower than that of trucks and Rail D, declining by 39.5% and 17.8%, respectively. At higher payloads (90 and 100%), the FC of rails was comparable but significantly lower than that of trucks. At the highest payload (100%), the percentage reduction in FC of Rail D and Rail E was 43% and 50%, respectively, compared to trucks. At all payload levels, CO_2_ emissions from trucks were significantly higher than those from rails. At 100% payload, CO_2_ emissions from Rail E were lower by 80.8% compared to trucks and by 64.9% compared to Rail D. At all payload levels, CO and VOC emissions from Rail D were higher than those from trucks and Rail E, and PM emissions from Rail E were lower than those of trucks and Rail D. At a payload range of 75–100%, N_2_O emissions from both rails were lower than from trucks. Thus, at all payloads, including 100% payload, Rail E had the lowest emissions of pollutants, except for CO.

**Table 3:** Variation of fuel consumption and pollutant emissions as affected by increasing payloads for three vehicle types.

	Payloads					
	10%	25%	50%	75%	90%	100%
**FC (L)**						
Trucks	39,785,695a	18,118,354a	11,167,297a	9,076,436a	8,469,946a	8,200,625a
Rail D	52,189,577a	20,682,038a	10,178,845a	6,677,770ab	5,511,375b	4,927,225b
Rail E	41,158,837a	16,464,641a	8,232,715a	5,488,740c	4,574,607b	4,116,752b
**CO**_**2**_ **(g/m**^**3**^)						
Trucks	246,742a	101,098a	52,550a	36,367a	30,973a	28,276a
Rail D	164,397b	65,148b	32,063b	21,035b	17,361b	15,521b
Rail E	54,329c	21,733c	10,867c	7,245c	6,038c	5,434c
**CO (g/m**^**3**^)						
Trucks	689.9b (3)	279.9b	143.2b	97.7b	82.5b	74.9b
Rail D	1,174.3a (1)	465.3a	229.0a	150.3a	124.0a	110.8a
Rail E	747.0b (2)	298.9b	149.4b	99.6b	83.0b	74.7b
**VOC (g/m**^**3**^)						
Trucks	321.6b	129.2b	65.1b	43.7b	36.6b	33.0b
Rail D	730.7a	289.5a	142.5a	93.5a	77.2a	69.0a
Rail E	305.6b	122.3b	61.1b	40.7b	34.0b	30.6c
**N**_**2**_**O (g/m**^**3**^)						
Trucks	3,296.9a	1,351.5a	703.0a	486.9a	414.8a	378.8a
Rail D	2,609.5a	1,034.1a	508.9a	333.9b	275.6b	246.4b
Rail E	1,494.1b	597.7b	298.9b	199.2b	166.1b	149.5b
**PM (g/m**^**3**^)						
Trucks	168.0a	68.2a	34.9a	23.9a	20.1a	18.3a
Rail D	182.7a	72.4a	35.6a	23.4a	19.3a	17.3a
Rail E	95.1b	38.3b	19.0b	12.1b	10.6b	9.5b

FC: fuel consumption, Rail D: diesel rail, Rail E: electric rail. Means followed by different letters within the same column show a statistically significant difference at *P value* <0.05 according to Duncan’s Multiple Range test

Significant regressions (*P value*< 0.05, R^2^ > 0.9) (Table 4) correlated FC and pollutant emissions of the three vehicle types with the payload factor through a cubic relationship.

**Table 4:** Significant correlations between fuel consumption (FC) and pollutant emissions (as dependant variables) and payload (as predictor) for different vehicle types.

				Unstandardised coefficients B				
	df	F	Sig.	constant	payload	payload**2	payload**3	*R* ^*2*^
FCTruck	3, 14	59.895	0.000	58254313.2	-2252717.6	33286.0	-158.5	0.916
FCRailD	3, 14	70.948	0.000	78991594.2	-3264876.7	47952.6	-228.3	0.938
FCRail E	3, 14	68.899	0.000	62165072.2	-2558865.7	37583.2	-178.9	0.924
CO2Truck	3, 14	67.156	0.000	370633.6	-15091.8	221.7	-1.05	0.935
CO2RailD	3, 14	70.948	0.000	248823.5	-10284.4	151.1	33.2	0.938
CO2RailE	3,14	69.899	0.000	82057.9	-3377.7	49.6	-0.2	0.937
COTruck	3,14	68.327	0.000	1038.6	-42.5	0.6	-0.003	0.936
CORailD	3,14	70.947	0.000	1777.4	-73.5	1.1	-0.005	0.938
CORail E	3,14	69.901	0.000	1128.3	-46.4	0.7	-0.003	0.937
VOCTruck	3,14	69.429	0.000	485.2	-19.9	0.3	-0.001	0.937
VOC RailD	3,14	70.932	0.000	1105.9	-45.7	0.7	-0.003	0.938
VOC RailE	3,14	69.906	0.000	461.7	-19.0	0.3	-0.001	0.937
N_2_OTruck	3,14	67.009	0.000	4951.7	-201.6	3.0	-0.014	0.935
N_2_ORailD	3,14	70.944	0.000	3949.6	-163.2	2.4	-0.011	0.938
N_2_ORailE	3,14	69.899	0.000	2256.6	-92.9	1.4	-0.006	0.937
PMTruck	3,14	68.197	0.000	252.8	-10.3	0.2	-0.001	0.936
PMRailD	3,14	79.964	0.000	276.5	-11.4	0.2	-0.001	0.938
PMRailE	3,14	69.955	0.000	143.6	-5.9	0.01	0.00	0.937

FC: fuel consumption, Rail D: diesel rail, Rail E: electric rail

**Fig 1:**
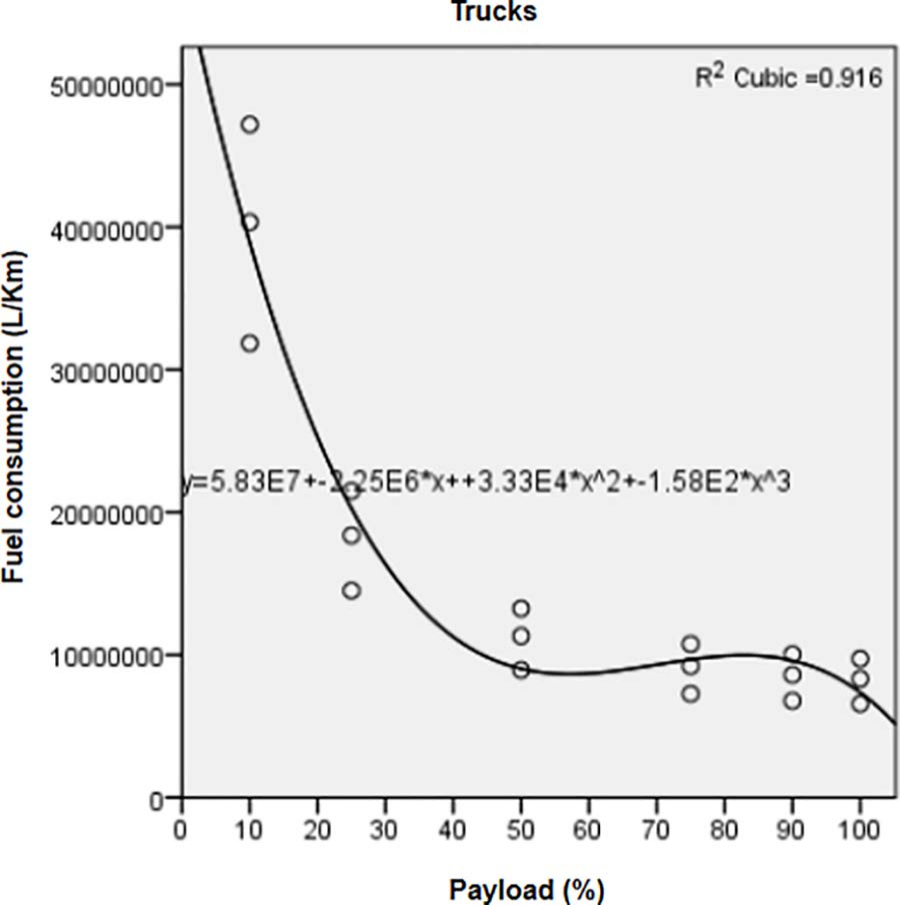
Correlation between fuel consumption and increasing payloads for trucks.

**Fig 2:**
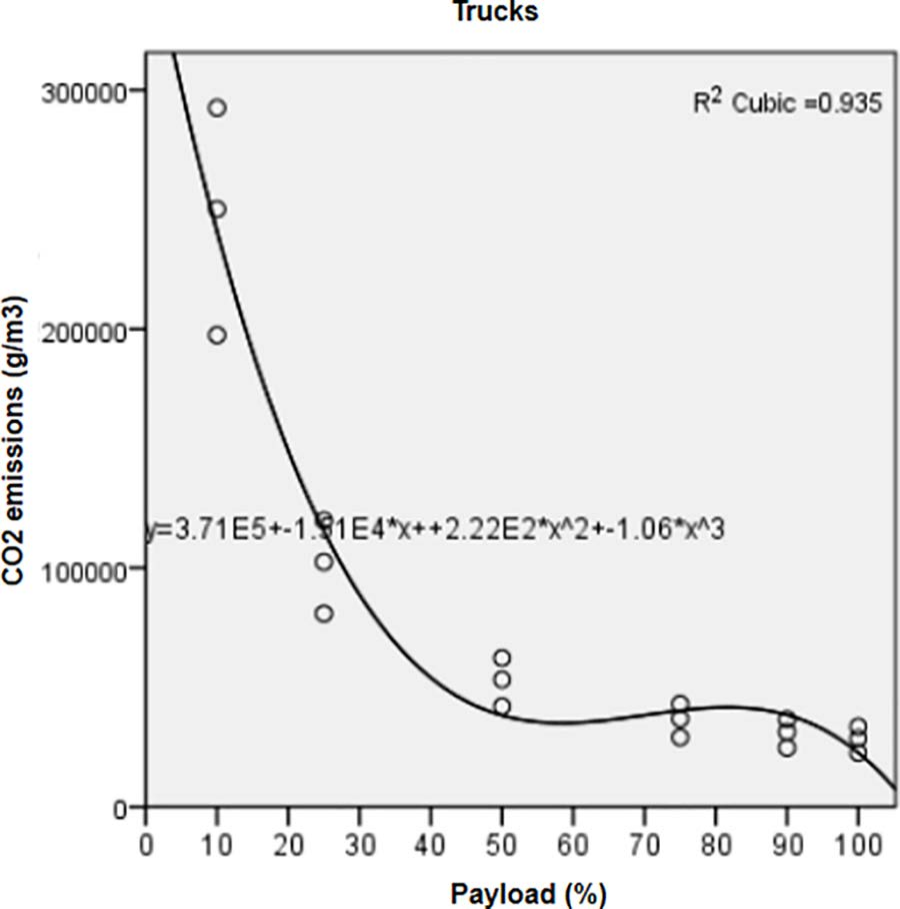
Correlation between CO_2_ emissions and increasing payloads for trucks.

**Fig 3:**
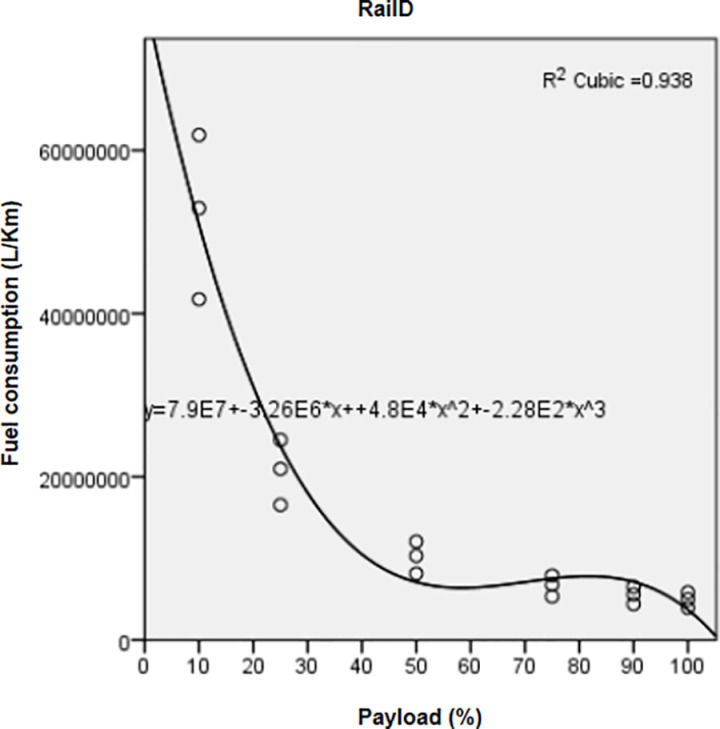
Correlation between fuel consumption and increasing payloads for Rail D.

**Fig 4:**
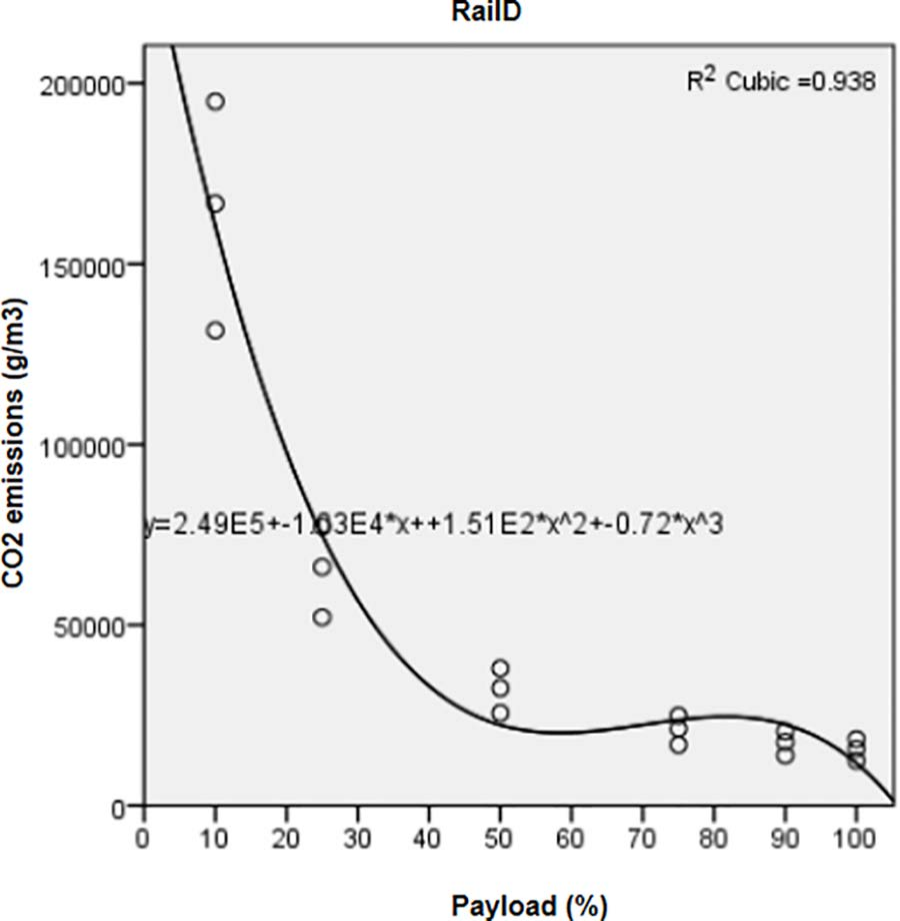
Correlation between CO_2_ emissions and increasing payloads for Rail D.

Regression curves represented for FC (Fig 1) and CO_2_ of trucks (Fig 2) showed that both indicators recorded the highest means at the lowest payloads. Mean values decreased from 10% to 50% payload, then increased from 50% to 90% payload; however, they were still lower than those recorded at 10–50% payload. They reached their minimal values at full payload (100%). Variations of FC (Fig 3) and CO_2_ from rails (Fig 4) followed a similar pattern as those of trucks.

Loading rates of vehicles play a critical role in determining the energy demand per tonne-kilometre for freight modes [72]. Guiggiani [73] explained that the engine must run with higher power requirements when the payload is high, which increases fuel consumption. A vehicle’s shape changes when it is loaded, changing the airflow around it. The drag force of the vehicle increases as more cargo creates more surface area. The weight of the cargo may compress the suspension, reducing ground clearance and increasing the frontal surface exposed to the air. As load weight increases, aerodynamic drag rises, increasing fuel consumption and emissions. In this study, an increase in payload from 50 to 100% caused a 26.5% reduction in fuel consumption of large trucks (3 axles, 10 tires). Parajuli [49] noted a reduction in fuel consumption by 35% and 32.2% for B-double trucks and six-axle articulated trucks, respectively, because of a payload increase from 58 to 98%. Also, more recently Baráth et al. [74] reported an increase in fuel consumption of diesel trucks because of increased load. Similarly to our findings, Coyle [75] reported a non-linear relationship between fuel consumption and payload of freight trucks.

Besides, CO_2_ levels recorded from the three vehicle types at payloads ≤50% exceeded the PEL indicated by the FSIS-ESHG [66]. While CO_2_ emissions from trucks and Rail D continued to be greater at payloads >50%, Rail E’s emissions were lower than this threshold. Therefore, utilizing the electric rails at full load capacity offers a notable benefit over the other two vehicle types: a reduction in fuel consumption of 43–50% and a reduction in CO_2_ emissions of 64.9–80.8%. Furthermore, at 100% payload, Rail E offers the advantage of decreasing CO and N_2_O emissions to be lower than the allowable limits [67,68].

### Effect of speed

With respect to the effect of varying speeds on fuel consumption (Table 5), it was found that at speeds lower than 60 km/hr, the FC of trucks was significantly higher than that of rails. With an average speed of 60 km/h, which is what most of the stretches will be traveling at, FC is reduced by about 40% with Rail D and by 50% with Rail E in comparison to trucks. Furthermore, at speeds lower than 60 km/hr, CO_2_ levels obtained from rails were significantly lower than those of trucks, and at speeds ≽ 60 km/hr, they were the lowest from Rail E compared with Rail D and trucks. At an average speed of 60 km/hr, CO_2_ levels were reduced by 45.1% and 80.7% from Rail E compared to Rail D and trucks, respectively. At speeds ≽60 km/hr, both CO and VOC emissions from Rail D significantly exceeded those from Rail E and trucks. At speeds ≤60 km/hr, N_2_O and PM emissions from rails (Rail D and Rail E) were significantly lower than from trucks, and at speeds > 60 km/hr, emissions of both pollutants from rail E were significantly lower than those from rail D. At the highest speeds (80 and 90 km/hr), N_2_O and PM emissions were comparable between trucks and Rail E.

**Table 5:** Variation of fuel consumption and pollutant emissions as affected by increasing speeds for three vehicle types.

	Speeds						
	30 Km/hr	40 Km/hr	50 Km/hr	60 Km/hr	70 Km/hr	80 Km/hr	90 Km/hr
**FC (L)**							
Truck	84,131,06a	83,068,65a	82,006,25a	82,006,25a	82,006,25a	83,068,65a	83,599,85ab
Rail D	14,490,18b	23,682,02b	35,276,04b	49,272,25b	65,670,66ab	84,471,26a	105,674,04a
Rail E	26,567,73b	30,352,86b	35,219,46b	41,167,52b	48,197,05b	56,310,45a	65,822,87b
**CO**_**2**_ **(g/m**^**3**^)							
Truck	40,448a	35,021a	30,947a	28,276a	27,190a	27,913a	30,684a
Rail D	4,564b	7,460b	11,112b	15,521b	20,686a	26,608a	33,287a
Rail E	3,507b	4,007b	4,649b	5,434c	6,362b	7,433b	8,689b
**CO (g/m**^**3**^)							
Truck	108.5a	90.1a	80.3a	74.9b	72.0b	70.6b	70.3b
Rail D	32.6b	53.3b	79.4a	110.8a	147.8a	190.1a	237.8a
Rail E	48.2b	55.1b	63.9a	74.7b	87.5b	102.2b	119.5b
**VOC (g/m**^**3**^)							
Truck	58.8a	45.9a	38.2ab	33.0b	29.4b	26.6b	24.5b
Rail D	20.3b	33.1ab	49.4a	69.0a	91.9a	118.2a	147.9a
Rail E	19.7b	22.5b	26.1b	30.6b	35.8b	41.8b	48.9b
**N**_**2**_**O(g/m**^**3**^)							
Truck	562.6a	472.1a	416.4a	378.8a	351.7a	331.2ab	315.2b
Rail D	72.5b	118.4b	176.4b	246.4b	328.4a	422.4a	528.4a
Rail E	96.4b	110.2b	127.9b	149.5b	174.9b	204.4b	238.9b
**PM (g/m**^**3**^)							
Truck	28.4a	23.4a	20.3a	18.3a	16.9ab	15.8b	14.9b
Rail D	5.1b	8.3b	12.3b	17.3a	23.0a	29.6a	37.0a
Rail E	6.1b	7.0b	8.2b	9.5b	11.1b	13.0b	15.2b

FC: fuel consumption, Rail D: diesel rail, Rail E: electric rail. Means followed by different letters within the same column show a statistically significant difference at *P value*<0.05 according to Duncan’s Multiple Range test

For trucks, FC did not significantly vary with increasing speeds, while on the contrary, for rails it did. In fact, regression analysis (Table 6) showed strong positive correlations between FC and speed for Rail D and Rail E (*R*^*2*^ = 0.91 and *R*^*2*^ = 0.77, respectively); the higher the speed, the higher the FC from the rails. Also, for both vehicles, there were significant positive correlations between emissions of all pollutants and speed (*R*^*2*^ = 0.91 for Rail D and *R*^*2*^* *= 0.77 for Rail E). Overall, emissions of all pollutants from rails increased with increasing speeds. On the contrary, emissions of VOC, N_2_O, and PM from trucks were significantly correlated with speed through a negative regression equation (*R*^*2*^ = 0.78, 0.61, and 0.66 for VOC, N_2_O, and PM, respectively); after recording the highest values at the lowest speed, emissions of these pollutants decreased progressively to reach their lowest values at the highest speed.

**Table 6:** Significant correlations between fuel consumption (FC) and pollutant emissions (as dependant variables) and speed (as predictor) for different vehicle types.

				Unstandardised coefficients B				
	*df*	*F*	*Sig.*	constant	speed	speed**2	speed**3	*R* ^*2*^
FCRail D	3,17	55.500	0.000	132783.852	7841.614	1201.096	-9.259E-7	0.91
FCRail E	3,17	19.294	0.000	2060682.25	7021.147	399.941	0.889	0.77
CO2Rail D	3,17	55.500	0.000	418.660	24.680	3.784	-1.852E-6	0.91
CO2Rail E	3,17	19.294	0.000	2720.128	9.266	0.528	0.001	0.77
CORail D	3,17	55.521	0.000	3.414	0.154	0.027	-1.852E-6	0.91
CORail E	3,17	19.311	0.000	37.484	0.123	0.007	1.574E-5	0.77
VOCTruck	3,17	19.677	0.000	129.952	-3.449	0.041	0.000	0.78
VOCRail D	3,17	55.472	0.000	1.847	0.111	0.017	7.506E-17	0.91
VOCRail E	3,17	19.317	0.000	15.406	0.047	0.003	6.481E-6	0.77
VOCTruck	3,17	8.904	0.001	1056.075	-23.740	0.277	-0.001	0.61
VOCRail D	3,17	55.496	0.000	6.872	0.380	0.060	-9.259E-7	0.91
VOCRail E	3,17	19.303	0.000	74.708	0.259	0.014	3.241E-5	0.77
PMTruck	3,17	10.953	0.000	55.946	-1.330	0.016	-6.574E-5	0.66
PMRail D	3,17	55.431	0.000	0.733	0.010	0.005	-1.852E-6	0.91
PMRail E	3,17	19.255	0.000	4.602	0.025	0.001	2.778E-6	0.77

FC: fuel consumption, Rail D: diesel rail, Rail E: electric rail

Fuel consumption of freight vehicles is sensitive to variations in speed because these vehicles require more engine power to overcome growing driving resistances (such as air drag) caused by high vehicle weight [41]. Contrarily to our findings, Fan et al. [76] found that fuel consumption rate (FCR) for trucks weighing 45–55 tons increased with increasing speeds.

Train resistance forces may also increase with the train length and weight as well as with the payload [77]. According to Frey and Graver [78], the train’s efficiency decreases when the locomotive is running at the higher power levels and throttle notches needed to sustain faster speeds. In the early work of Fullerton and Dick [40] conducted on rails, it was found that speed has a reverse-parabolic reaction, which means that both a sudden increase and a decrease in speed have a negative impact on fuel efficiency and raise fuel consumption. According to Givoni et al. [18] the basic laws of physics suggest that the fuel consumption would grow nonlinearly at operating speeds greater than 100 km/h, primarily because of disproportionately higher aerodynamic drag. However, the relationship between operating speed and energy consumption is complex, and data on energy consumption for a specific train and route at different operating speeds and station spacings is lacking. This makes comparing the results of the current study with those of similar research challenging. Adding that data on the energy use in railways is a main challenge that is still to overcome [79].

According to the most recent analysis by Bulakh [80], railway trains moving at 90 km/h on a straight track segment might use 18.9% more energy. However, the energy consumption estimation model took into account a number of additional factors that were not examined in this study, including section length, travel time, and train mass.

In this study, when the speed increased from 60 to 90 km/h, CO_2_ emissions increased by 7.84%, 53.4%, and 37.4% from trucks, Rail D, and Rail E, respectively. Lower emissions from trucks traveling at higher speeds may be linked to less substantial changes in FC, but higher emissions from railroads with rising speeds may be linked to the significant increase in FC. In the early study of Gao et al. [81], fuel consumption and carbon emissions rose from roughly 33% to 38% when the speed increased from 80 to 120 km/h. Thus, it is recommended to take carbon emissions into account when determining the speed limit on the studied corridor. Further, it is generally recognized that while a vehicle is traveling between 50 and 80 km/h, CO_2_ emissions will be at a relatively low level [61]. At 50 and 80 km/h, trucks’ CO_2_ levels were at their lowest. Nevertheless, at all investigated speeds, the CO_2_ emissions from trucks and Rail D were higher than the PEL [66], but those from Rail E were lower starting at 60–90 km/h. Consequently, employing Rail E guarantees that CO_2_ emissions will remain within allowable levels even when traveling at speeds of up to 90 km/h. VOC and PM emissions from Rail E exceed the authorized limits [69,70] at all speeds, whereas CO and N_2_O emissions were below the permitted levels [67,68] at speeds below 70 km/h. In their investigation of rail transport emissions, Zhao et al. [82] concluded that electric locomotives produced a notably lower amount of particulate matter than diesel trains. They attributed this finding to the smoother running of electric vehicles and to their regenerative braking, which reduces mechanical wear and the related PM production.

### Effect of road gradient and curvature

**Fig 5:**
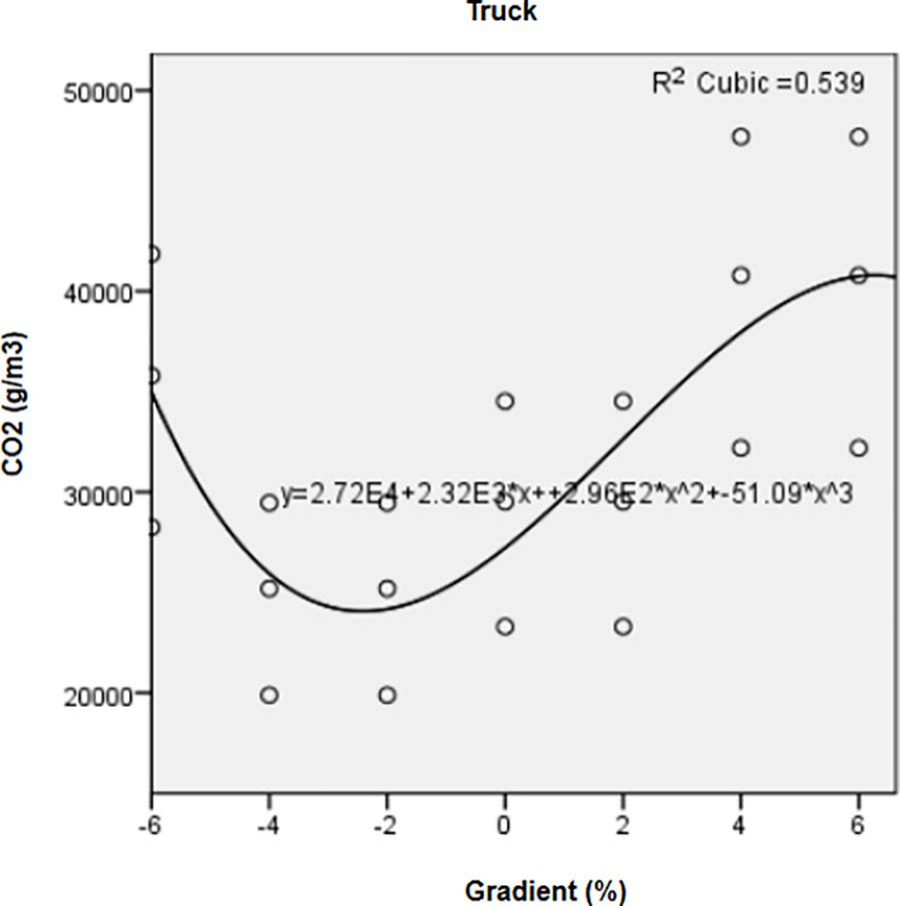
Regression curve showing the correlation between CO_2_ emissions and increasing gradient for trucks.

Road gradient was correlated with CO_2_ emissions of trucks through a significant cubic regression (Fig 5) showing two turning points: a decrease from a gradient of -6% to -2%, followed by an increase from -2–6%. Although statistically significant (*R2* = 0.54), the model describes only 50% of the variations of CO_2_ as affected by increasing gradient. In this study, payload and gradient had a greater impact on truck CO_2_ emissions than speed. These findings are in line with those of Posada-Henao et al. [83], who found that emissions from similar truck types (3 axles) were significantly influenced by the roadway slope rather than speed.

Gradient had no significant effect on the FC of trucks. On the contrary, Saptarini et al. [39] found that in unloaded conditions, fuel consumption of coal mine transport trucks increased because of an addition of 1% road slope. According to Joumard [84], road gradient affects traction resistance, which in turn affects fuel consumption and emissions. Driving downhill lessens emissions, while driving uphill raises them; however, the decrease does not completely counteract the increase. Heavy-duty trucks are more affected by this pattern because of their heavier mass.

On the other hand, the variations of FC and emissions of Rail D were positively and linearly correlated with the road gradient (*R*^*2*^ = 0.84) (**Table 7**). Thus, the higher the road gradient, the higher the fuel consumption and consequently pollutant emissions from Rail D. Fuel consumption depends on the forces associated with the uphill and downhill slopes, which in turn affect the emissions of the railway [85]. Earlier, Tolliver et al. [86] found that grade is one of the most important factors affecting train fuel consumption. The preferred maximum design grade for a freight railroad is 1% [54]. Jordan’s current railway has 2.5% grades because of the topographic conditions and to avoid the difficulties of constructing bridges, which could be expensive to build and maintain. This might explain the significant effect of gradient on diesel rails. The electric rails’ FC variations showed to be more significantly impacted by the speed and payload than by the road gradient and curvature.

**Table 7:** Correlation between fuel consumption (FC) and pollutant emissions (as dependant variables) with road gradient and curvature (as predictors) for diesel rail.

				Unstandardised coefficients B			
Dep.	*df*	*F*	*Sig.*	constant	gradient		*R* ^*2*^
FCRailD	1,19	101.5	0.000	5592260.89	1458692.61		0.84
CO2RailD	1,19	101.5	0.000	17615.62	4594.88		0.84
CO RailD	1,19	101.5	0.000	125.826	32.820		0.84
VOCRailD	1,19	101.4	0.000	78.291	20.422		0.84
N_2_ORailD	1,19	101.5	0.000	279.612	72.935		0.84
PMRailD	1,19	101.4	0.000	19.573	5.105		0.84
**Dep.**	***df***	***F***	***Sig.***	**constant**	**curvature**	curvature **2	***R*** ^***2***^
FCRailD	2,18	6.239	0.009	7106491.3	-2276.012	0.256	0.41
CO_2_RailD	2,18	6.239	0.009	22385.5	-7.169	0.001	0.41
CORailD	2,18	6.239	0.009	159.9	-0.051	5.764E-6	0.41
VOCRailD	2,18	6.239	0.009	99.491	-0.032	3.587E-6	0.41
N_2_ORailD	2,18	6.239	0.009	355.324	-0.114	1.281E-5	0.41
PMRailD	2,18	6.238	0.009	24.871	-0.008	8.963E-7	0.41

Furthermore, correlations between the factor ‘curvature’ with FC and emissions of Rail D were weak (*R*^*2*^ = 0.41) (Table 7) showing a minor effect of this factor compared to other factors such as payload, speed, and gradient. On the contrary, Bakibillah et al. [42] pointed out that driving on horizontal curves induces more fuel consumption and emissions because sometimes the driver needs to break and accelerate.

## Conclusion

Based on the study findings, Jordan needs to upgrade its railway system or establish a new line for phosphate transportation to enhance efficiency, reduce fuel consumption, and minimize truck-related pollution. Compared to trucks, railways showed to be less polluting, with the electric railways being the best option. Their use might substantially reduce CO_2_ emissions compared with trucks and diesel trains. Although requiring less maintenance and having lower operating costs, transitioning from trucks to railways, particularly electric rails, is connected with higher capital expenditure (CAPEX), a substantial number of structures required, and the challenges posed by such investments. The study compared phosphate transport using trucks and diesel railways with electric railways, assuming conventional diesel generators. Future studies might focus on determining pollution levels using electricity generated from cleaner sources such as biodiesel or renewable green fuel. This type of diesel has been proved to be more environmentally friendly, emitting less carbon dioxide and nitrogen oxide than petroleum diesel. As a result, our findings might be updated to reflect the use of this diesel, which holds potential for better results without the need for significant investments in alternative energy sources such as solar, wind, hydroelectric power, or hydrogen. Nevertheless, given Jordan’s current diesel fleet and the high cost of investment in upgrading railways and the time needed to implement such a development strategy, the Jordan Phosphate Mine Company and the Jordanian Ministry of Transport authorities may consider using a maximum payload and maintaining a consistent average speed of 50–70 km/h for trucks and 60 km/h for trains as an initiative adaptation strategy to reduce fuel consumption and pollutant emissions from existing freight transport modes.

## Supporting information

S1 Raw Data (DOCX)
