# Supplementary material for: Electric trains for reduction of fuel consumption and emissions from the freight transport in Jordan
Source: PLoS One. 2025 May 9;20(5):e0323121. doi: 10.1371/journal.pone.0323121 (PMC12063814; doi:10.1371/journal.pone.0323121)
Supplement: S1 Raw Data — (DOCX) [file pone.0323121.s001.docx]

**Raw Data Speed**

| **Year** | **Vehicle** | **Speed** | FC truck | FC Rail D | FC Rail E | CO2 truck | CO2 Rail D | CO2 Rail E | CO truck | CO Rail D | CO Rail E |
| --- | --- | --- | --- | --- | --- | --- | --- | --- | --- | --- | --- |
| **2020** | **1** | **30** | 6732518 | 1159493 | 2125928 | 32367.9 | 3652.4 | 2806.2 | 86.8 | 26.1 | 38.6 |
| **2021** | **1** | **30** | 8530849 | 1469580 | 2694472 | 41013.7 | 4629.2 | 3556.7 | 110 | 33.1 | 48.9 |
| **2022** | **1** | **30** | 9975950 | 1717983 | 3149919 | 47961.3 | 5411.7 | 4157.9 | 128.6 | 38.7 | 57.2 |
| **2020** | **1** | **40** | 6647500 | 1895015 | 2428811 | 28024.6 | 5969.3 | 3206 | 72.1 | 42.6 | 44.1 |
| **2021** | **1** | **40** | 8423122 | 2401806 | 3078356 | 35510.3 | 7565.7 | 4063.4 | 91.3 | 54 | 55.9 |
| **2022** | **1** | **40** | 9849974 | 2807784 | 3598691 | 41525.6 | 8844.5 | 4750.3 | 106.8 | 63.2 | 65.3 |
| **2020** | **1** | **50** | 6562482 | 2822760 | 2818232 | 24765.3 | 8891.7 | 3720.1 | 64.3 | 63.5 | 51.2 |
| **2021** | **1** | **50** | 8315394 | 3577660 | 3571922 | 31380.4 | 11269.6 | 4714.9 | 81.4 | 80.5 | 64.8 |
| **2022** | **1** | **50** | 9723998 | 4182392 | 4175684 | 36696.1 | 13174.5 | 5511.9 | 95.2 | 94.1 | 75.8 |
| **2020** | **1** | **60** | 6562482 | 3942725 | 3294191 | 22627.8 | 12419.6 | 4348.3 | 59.9 | 88.7 | 59.8 |
| **2021** | **1** | **60** | 8315394 | 4997142 | 4175168 | 28672 | 15741 | 5511.2 | 75.9 | 112.4 | 75.8 |
| **2022** | **1** | **60** | 9723998 | 5841809 | 4880897 | 33528.9 | 18401.7 | 6442.8 | 88.8 | 131.4 | 88.6 |
| **2020** | **1** | **70** | 6562482 | 5254912 | 3856688 | 21758.6 | 16553 | 5090.8 | 57.6 | 118.2 | 70 |
| **2021** | **1** | **70** | 8315394 | 6660252 | 4888096 | 27570.6 | 20979.8 | 6452.3 | 73 | 149.9 | 88.7 |
| **2022** | **1** | **70** | 9723998 | 7786034 | 5714331 | 32241 | 24526 | 7542.9 | 85.4 | 175.2 | 103.7 |
| **2020** | **1** | **80** | 6647500 | 6759320 | 4505916 | 22336.9 | 21291.9 | 5947.8 | 56.5 | 152.1 | 81.8 |
| **2021** | **1** | **80** | 8423122 | 8566989 | 5710949 | 28303.4 | 26986 | 7538.5 | 71.6 | 192.8 | 103.7 |
| **2022** | **1** | **80** | 9849974 | 10015068 | 6676271 | 33097.9 | 31547.5 | 8812.7 | 83.8 | 225.3 | 121.2 |
| **2020** | **1** | **90** | 6690009 | 8455950 | 5267092 | 24554.4 | 26636.2 | 6952.6 | 56.3 | 190.3 | 95.6 |
| **2021** | **1** | **90** | 8476985 | 10717354 | 6675689 | 31113.2 | 33759.7 | 8811.9 | 71.3 | 241.1 | 121.2 |
| **2022** | **1** | **90** | 9912962 | 12528909 | 7804081 | 36383.7 | 39466.1 | 10301.4 | 83.4 | 281.9 | 141.6 |

| **Year** | **Vehicle** | **Speed** | VOC truck | VOC Rail D | VOC Rail E | NO2 truck | NO2 Rail D | NO2 Rail E | PM truck | PM Rail D | PM Rail E |
| --- | --- | --- | --- | --- | --- | --- | --- | --- | --- | --- | --- |
| **2020** | **1** | **30** | 47 | 16.2 | 15.8 | 450.2 | 58 | 77.2 | 22.7 | 4.1 | 4.9 |
| **2021** | **1** | **30** | 59.6 | 20.6 | 20 | 570.5 | 73.5 | 97.8 | 28.8 | 5.1 | 6.2 |
| **2022** | **1** | **30** | 69.7 | 24.1 | 23.4 | 667.1 | 85.9 | 114.3 | 33.7 | 6 | 7.3 |
| **2020** | **1** | **40** | 36.7 | 26.5 | 18 | 377.8 | 94.8 | 88.2 | 18.7 | 6.6 | 5.6 |
| **2021** | **1** | **40** | 46.5 | 33.6 | 22.9 | 478.7 | 120.1 | 111.7 | 23.7 | 8.4 | 7.1 |
| **2022** | **1** | **40** | 54.4 | 39.3 | 26.7 | 559.7 | 140.4 | 130.6 | 27.7 | 9.8 | 8.3 |
| **2020** | **1** | **50** | 30.5 | 39.5 | 20.9 | 333.2 | 141.1 | 102.3 | 16.3 | 9.9 | 6.5 |
| **2021** | **1** | **50** | 38.7 | 50.1 | 26.5 | 422.3 | 178.9 | 129.7 | 20.6 | 12.5 | 8.3 |
| **2022** | **1** | **50** | 45.3 | 58.6 | 31 | 493.8 | 209.1 | 151.6 | 24.1 | 14.6 | 9.7 |
| **2020** | **1** | **60** | 26.4 | 55.2 | 24.5 | 303.1 | 197.1 | 119.6 | 14.7 | 13.8 | 7.6 |
| **2021** | **1** | **60** | 33.5 | 70 | 31 | 384.1 | 249.9 | 151.6 | 18.6 | 17.5 | 9.6 |
| **2022** | **1** | **60** | 39.2 | 81.8 | 36.2 | 449.2 | 292.1 | 177.2 | 21.7 | 20.5 | 11.3 |
| **2020** | **1** | **70** | 23.5 | 73.6 | 28.6 | 281.4 | 262.8 | 140 | 13.5 | 18.4 | 8.9 |
| **2021** | **1** | **70** | 29.8 | 93.2 | 36.3 | 356.6 | 333 | 177.4 | 17.1 | 23.3 | 11.3 |
| **2022** | **1** | **70** | 34.8 | 109 | 42.4 | 417 | 389.3 | 207.4 | 20 | 27.3 | 13.2 |
| **2020** | **1** | **80** | 21.3 | 94.6 | 33.5 | 265 | 338 | 163.6 | 12.6 | 23.7 | 10.4 |
| **2021** | **1** | **80** | 27 | 119.9 | 42.4 | 335.8 | 428.4 | 207.3 | 16 | 30 | 13.2 |
| **2022** | **1** | **80** | 31.5 | 140.2 | 49.6 | 392.7 | 500.8 | 242.4 | 18.7 | 35.1 | 15.4 |
| **2020** | **1** | **90** | 19.6 | 118.4 | 39.1 | 252.2 | 422.8 | 191.2 | 12 | 29.6 | 12.2 |
| **2021** | **1** | **90** | 24.8 | 150 | 49.6 | 319.6 | 535.9 | 242.3 | 15.2 | 37.5 | 15.4 |
| **2022** | **1** | **90** | 29 | 175.4 | 58 | 373.7 | 626.5 | 283.3 | 17.7 | 43.9 | 18 |

**Raw Data Payload**

| Year | Payload | FC truck | FC Rail D | FC Rail E | CO2 truck | CO2 Rail D | CO2 Rail E | CO truck | CO Rail D | CO Rail E |
| --- | --- | --- | --- | --- | --- | --- | --- | --- | --- | --- |
| 2020 | 10 | 31838173.6 | 41764474 | 32937174.2 | 197453.6 | 131558.1 | 43477.1 | 552.1 | 939.7 | 597.8 |
| 2021 | 10 | 40342504.6 | 52920285 | 41735103.6 | 250195.6 | 166698.9 | 55090.3 | 699.5 | 1190.7 | 757.5 |
| 2022 | 10 | 47176406.9 | 61883972 | 48804234 | 292578 | 194934.5 | 64421.6 | 818 | 1392.4 | 885.8 |
| 2020 | 25 | 14499062.7 | 16551977 | 13176764.3 | 80903.1 | 52138.7 | 17393.3 | 224 | 372.4 | 239.2 |
| 2021 | 25 | 18371923.9 | 20969605 | 16693567.8 | 102513.2 | 66054.3 | 22035.5 | 283.8 | 471.8 | 303 |
| 2022 | 25 | 21484074.1 | 24524532 | 19523588.2 | 119878.6 | 77252.3 | 25771.1 | 331.9 | 551.8 | 354.4 |
| 2020 | 50 | 8936536.8 | 8145809 | 6588382.1 | 42052.9 | 25659.3 | 8696.7 | 114.6 | 183.3 | 119.6 |
| 2021 | 50 | 11323585.3 | 10321343 | 8347968 | 53285.7 | 32512.2 | 11019.3 | 145.2 | 232.2 | 151.5 |
| 2022 | 50 | 13241767.6 | 12069383 | 9761794.1 | 62312.2 | 38018.6 | 12885.6 | 169.8 | 271.6 | 177.2 |
| 2020 | 75 | 7263342.9 | 5344713.5 | 4393044.2 | 29102.9 | 16835.9 | 5798.8 | 78.2 | 120.3 | 79.7 |
| 2021 | 75 | 9203462.7 | 6770930.9 | 5565312 | 36876.6 | 21328.4 | 7346.2 | 99 | 152.4 | 101 |
| 2022 | 75 | 10762502.4 | 7917667.2 | 6507862.7 | 43123.4 | 24940.7 | 8590.4 | 115.8 | 178.2 | 118.1 |
| 2020 | 90 | 6778004.2 | 4411002.5 | 3661264.8 | 24786.2 | 13894.7 | 4832.9 | 66 | 99.3 | 66.5 |
| 2021 | 90 | 8588484.6 | 5589362.2 | 4639338.8 | 31406.9 | 17606.5 | 6123.9 | 83.6 | 125.8 | 84.2 |
| 2022 | 90 | 10043348.8 | 6533761.8 | 5423218.9 | 36727.1 | 20581.4 | 7158.7 | 97.8 | 147 | 98.4 |
| 2020 | 100 | 6562482 | 3942725.1 | 3294191.1 | 22627.8 | 12419.6 | 4348.3 | 59.9 | 88.7 | 59.8 |
| 2021 | 100 | 8315394.1 | 4997141.9 | 4175168.1 | 28672 | 15741 | 5511.2 | 75.9 | 112.4 | 75.8 |
| 2022 | 100 | 9723997.6 | 5841809.1 | 4880897 | 33528.9 | 18401.7 | 6442.8 | 88.8 | 131.4 | 88.6 |

| Year | Payload | VOC truck | VOC Rail D | VOC Rail E | NO2 truck | NO2 Rail D | NO2 Rail E | PM truck | PM Rail D | PM Rail E |
| --- | --- | --- | --- | --- | --- | --- | --- | --- | --- | --- |
| 2020 | 10 | 257.4 | 584.7 | 244.6 | 2638.3 | 2088.2 | 1195.6 | 134.4 | 146.2 | 76.1 |
| 2021 | 10 | 326.1 | 740.9 | 309.9 | 3343 | 2646 | 1515 | 170.3 | 185.2 | 96.4 |
| 2022 | 10 | 381.3 | 866.4 | 362.4 | 3909.3 | 3094.2 | 1771.6 | 199.2 | 216.6 | 112.7 |
| 2020 | 25 | 103.4 | 231.7 | 97.8 | 1081.5 | 827.6 | 478.3 | 54.6 | 57.9 | 30.4 |
| 2021 | 25 | 131 | 293.6 | 124 | 1370.4 | 1048.5 | 606 | 69.2 | 73.4 | 38.6 |
| 2022 | 25 | 153.2 | 343.3 | 145 | 1602.5 | 1226.2 | 708.7 | 80.9 | 85.8 | 45.1 |
| 2020 | 50 | 52.1 | 114 | 48.9 | 562.6 | 407.3 | 239.2 | 28 | 28.5 | 15.2 |
| 2021 | 50 | 66 | 144.5 | 62 | 712.9 | 516.1 | 303 | 35.4 | 36.1 | 19.3 |
| 2022 | 50 | 77.2 | 169 | 72.5 | 833.6 | 603.5 | 354.4 | 41.4 | 42.2 | 22.6 |
| 2020 | 75 | 35 | 74.8 | 32.6 | 389.6 | 267.2 | 159.5 | 19.1 | 18.7 | 10.2 |
| 2021 | 75 | 44.3 | 94.8 | 41.3 | 493.7 | 338.6 | 202 | 24.2 | 23.7 | 12.9 |
| 2022 | 75 | 51.8 | 110.9 | 48.3 | 577.3 | 395.9 | 236.2 | 28.3 | 27.7 | 15 |
| 2020 | 90 | 29.3 | 61.8 | 27.2 | 332 | 220.6 | 132.9 | 16.1 | 15.4 | 8.5 |
| 2021 | 90 | 37.1 | 78.3 | 34.5 | 420.6 | 279.5 | 168.4 | 20.4 | 19.6 | 10.7 |
| 2022 | 90 | 43.4 | 91.5 | 40.3 | 491.9 | 326.7 | 196.9 | 23.9 | 22.9 | 12.5 |
| 2020 | 100 | 26.4 | 55.2 | 24.5 | 303.1 | 197.1 | 119.6 | 14.7 | 13.8 | 7.6 |
| 2021 | 100 | 33.5 | 70 | 31 | 384.1 | 249.9 | 151.6 | 18.6 | 17.5 | 9.6 |
| 2022 | 100 | 39.2 | 81.8 | 36.2 | 449.2 | 292.1 | 177.2 | 21.7 | 20.5 | 11.3 |

**Raw Data Gradient**

**Rail**

| year | vehicle | gradient | gradient trail | FC Rail D | FC Rail E | CO2 Rail D | CO2 Rail E | CO Rail D | CO Rail E | VOC Rail D | VOC Rail E |
| --- | --- | --- | --- | --- | --- | --- | --- | --- | --- | --- | --- |
| 1 | 1 | 1 | -2.25 | 1848691.2 | 3294191 | 5823.4 | 4348.3 | 41.6 | 59.8 | 25.9 | 24.5 |
| 2 | 1 | 1 | -2.25 | 2343093.1 | 4175168 | 7380.7 | 5511.2 | 52.7 | 75.8 | 32.8 | 31 |
| 3 | 1 | 1 | -2.25 | 2739146.3 | 4880897 | 8628.3 | 6442.8 | 61.6 | 88.6 | 38.4 | 36.2 |
| 1 | 1 | 2 | -1.5 | 2723882.7 | 3294191 | 8580.2 | 4348.3 | 61.3 | 59.8 | 38.1 | 24.5 |
| 2 | 1 | 2 | -1.5 | 3452340.2 | 4175168 | 10874.9 | 5511.2 | 77.7 | 75.8 | 48.3 | 31 |
| 3 | 1 | 2 | -1.5 | 4035889.4 | 4880897 | 12713.1 | 6442.8 | 90.8 | 88.6 | 56.5 | 36.2 |
| 1 | 1 | 3 | -0.75 | 3599344.9 | 3294191 | 11337.9 | 4348.3 | 81 | 59.8 | 50.4 | 24.5 |
| 2 | 1 | 3 | -0.75 | 4561930.3 | 4175168 | 14370.1 | 5511.2 | 102.6 | 75.8 | 63.9 | 31 |
| 3 | 1 | 3 | -0.75 | 5333033.7 | 4880897 | 16799.1 | 6442.8 | 120 | 88.6 | 74.7 | 36.2 |
| 1 | 1 | 4 | 0 | 4474930.3 | 3294191 | 14096 | 4348.3 | 100.7 | 59.8 | 62.7 | 24.5 |
| 2 | 1 | 4 | 0 | 5671676.6 | 4175168 | 17865.8 | 5511.2 | 127.6 | 75.8 | 79.4 | 31 |
| 3 | 1 | 4 | 0 | 6630360.4 | 4880897 | 20885.6 | 6442.8 | 149.2 | 88.6 | 92.8 | 36.2 |
| 1 | 1 | 5 | 0.75 | 5350491 | 3294191 | 16854.1 | 4348.3 | 120.4 | 59.8 | 74.9 | 24.5 |
| 2 | 1 | 5 | 0.75 | 6781391.6 | 4175168 | 21361.4 | 5511.2 | 152.6 | 75.8 | 94.9 | 31 |
| 3 | 1 | 5 | 0.75 | 7927650.6 | 4880897 | 24972.1 | 6442.8 | 178.4 | 88.6 | 111 | 36.2 |
| 1 | 1 | 6 | 1.5 | 6225879.4 | 3294191 | 19611.5 | 4348.3 | 140.1 | 59.8 | 87.2 | 24.5 |
| 2 | 1 | 6 | 1.5 | 7890888.1 | 4175168 | 24856.3 | 5511.2 | 177.5 | 75.8 | 110.5 | 31 |
| 3 | 1 | 6 | 1.5 | 9224685.5 | 4880897 | 29057.8 | 6442.8 | 207.6 | 88.6 | 129.2 | 36.2 |
| 1 | 1 | 7 | 2.25 | 7100947.9 | 3294191 | 22368 | 4348.3 | 159.8 | 59.8 | 99.4 | 24.5 |
| 2 | 1 | 7 | 2.25 | 8999979.3 | 4175168 | 28349.9 | 5511.2 | 202.5 | 75.8 | 126 | 31 |
| 3 | 1 | 7 | 2.25 | 10521246.3 | 4880897 | 33141.9 | 6442.8 | 236.7 | 88.6 | 147.3 | 36.2 |

| year | vehicle | gradient | gradient trail | NO2 Rail D | NO2 Rail E | PM Rail D | PM Rail E |
| --- | --- | --- | --- | --- | --- | --- | --- |
| 1 | 1 | 1 | -2.25 | 92.4 | 119.6 | 6.5 | 7.6 |
| 2 | 1 | 1 | -2.25 | 117.2 | 151.6 | 8.2 | 9.6 |
| 3 | 1 | 1 | -2.25 | 137 | 177.2 | 9.6 | 11.3 |
| 1 | 1 | 2 | -1.5 | 136.2 | 119.6 | 9.5 | 7.6 |
| 2 | 1 | 2 | -1.5 | 172.6 | 151.6 | 12.1 | 9.6 |
| 3 | 1 | 2 | -1.5 | 201.8 | 177.2 | 14.1 | 11.3 |
| 1 | 1 | 3 | -0.75 | 180 | 119.6 | 12.6 | 7.6 |
| 2 | 1 | 3 | -0.75 | 228.1 | 151.6 | 16 | 9.6 |
| 3 | 1 | 3 | -0.75 | 266.7 | 177.2 | 18.7 | 11.3 |
| 1 | 1 | 4 | 0 | 223.8 | 119.6 | 15.7 | 7.6 |
| 2 | 1 | 4 | 0 | 283.6 | 151.6 | 19.9 | 9.6 |
| 3 | 1 | 4 | 0 | 331.5 | 177.2 | 23.2 | 11.3 |
| 1 | 1 | 5 | 0.75 | 267.5 | 119.6 | 18.7 | 7.6 |
| 2 | 1 | 5 | 0.75 | 339.1 | 151.6 | 23.7 | 9.6 |
| 3 | 1 | 5 | 0.75 | 396.4 | 177.2 | 27.8 | 11.3 |
| 1 | 1 | 6 | 1.5 | 311.3 | 119.6 | 21.8 | 7.6 |
| 2 | 1 | 6 | 1.5 | 394.5 | 151.6 | 27.6 | 9.6 |
| 3 | 1 | 6 | 1.5 | 461.2 | 177.2 | 32.3 | 11.3 |
| 1 | 1 | 7 | 2.25 | 355.1 | 119.6 | 24.9 | 7.6 |
| 2 | 1 | 7 | 2.25 | 450 | 151.6 | 31.5 | 9.6 |
| 3 | 1 | 7 | 2.25 | 526.1 | 177.2 | 36.8 | 11.3 |

**Raw Data Gradient**

**Truck**

| year | vehicle | gradient | gradient truck | FC truck | CO2truck | CO truck | VOC truck | NO2 truck | PM truck |
| --- | --- | --- | --- | --- | --- | --- | --- | --- | --- |
| 1 | 1 | 1 | -6 | 6844589.26 | 28241.9 | 68.3 | 29.9 | 351.7 | 18.1 |
| 2 | 1 | 1 | -6 | 8672855.32 | 35785.7 | 86.5 | 37.8 | 445.6 | 22.9 |
| 3 | 1 | 1 | -6 | 10142011.65 | 41847.7 | 101.2 | 44.2 | 521.1 | 26.8 |
| 1 | 1 | 2 | -4 | 6406214.23 | 19879.9 | 57.7 | 25.7 | 277.5 | 13.8 |
| 2 | 1 | 2 | -4 | 8117385.43 | 25190.1 | 73.1 | 32.6 | 351.6 | 17.5 |
| 3 | 1 | 2 | -4 | 9492446.79 | 29457.2 | 85.4 | 38.1 | 411.2 | 20.5 |
| 1 | 1 | 3 | -2 | 6443409.69 | 19879.9 | 57.7 | 25.7 | 277.5 | 13.8 |
| 2 | 1 | 3 | -2 | 8164516.21 | 25190.1 | 73.1 | 32.6 | 351.6 | 17.5 |
| 3 | 1 | 3 | -2 | 9547561.39 | 29457.2 | 85.4 | 38.1 | 411.2 | 20.5 |
| 1 | 1 | 4 | 0 | 6467321.05 | 23292.8 | 57.9 | 25.7 | 308.6 | 14 |
| 2 | 1 | 4 | 0 | 8194814.57 | 29514.6 | 73.4 | 32.5 | 391 | 17.7 |
| 3 | 1 | 4 | 0 | 9582992.2 | 34514.3 | 85.8 | 38 | 457.2 | 20.7 |
| 1 | 1 | 5 | 2 | 6591660.15 | 23292.8 | 57.9 | 25.7 | 308.6 | 14 |
| 2 | 1 | 5 | 2 | 8352366.02 | 29514.6 | 73.4 | 32.5 | 391 | 17.7 |
| 3 | 1 | 5 | 2 | 9767232.41 | 34514.3 | 85.8 | 38 | 457.2 | 20.7 |
| 1 | 1 | 6 | 4 | 6791452.89 | 32187.6 | 60.6 | 29.5 | 373.9 | 17.2 |
| 2 | 1 | 6 | 4 | 8605525.63 | 40785.2 | 76.7 | 37.4 | 473.8 | 21.8 |
| 3 | 1 | 6 | 4 | 10063276.52 | 47694.1 | 89.7 | 43.7 | 554.1 | 25.5 |
| 1 | 1 | 7 | 6 | 7790416.6 | 32187.6 | 60.6 | 29.5 | 373.9 | 17.2 |
| 2 | 1 | 7 | 6 | 9871323.68 | 40785.2 | 76.7 | 37.4 | 473.8 | 21.8 |
| 3 | 1 | 7 | 6 | 11543497.05 | 47694.1 | 89.7 | 43.7 | 554.1 | 25.5 |

**Raw Data Curvature**

| Year | Curvature | FC truck | FC Rail D | FC Rail E | CO2 truck | CO2 Rail D | CO2 Rail E | CO truck | CO Rail D | CO Rail E |
| --- | --- | --- | --- | --- | --- | --- | --- | --- | --- | --- |
| 2020 | 75 | 6806284.2 | 7017893 | 3294191 | 22627.8 | 22106.4 | 4348.3 | 59.9 | 157.9 | 59.8 |
| 2021 | 75 | 8624318.5 | 8894713 | 4175168 | 28672 | 28018.4 | 5511.2 | 75.9 | 200.1 | 75.8 |
| 2022 | 75 | 10085252.9 | 10398187 | 4880897 | 33528.9 | 32754.3 | 6442.8 | 88.8 | 234 | 88.6 |
| 2020 | 125 | 6806284.2 | 5611697 | 3294191 | 22627.8 | 17676.9 | 4348.3 | 59.9 | 126.3 | 59.8 |
| 2021 | 125 | 8624318.5 | 7112453 | 4175168 | 28672 | 22404.2 | 5511.2 | 75.9 | 160 | 75.8 |
| 2022 | 125 | 10085252.9 | 8314672 | 4880897 | 33528.9 | 26191.2 | 6442.8 | 88.8 | 187.1 | 88.6 |
| 2020 | 225 | 6487466 | 4674234 | 3294191 | 22627.8 | 14723.8 | 4348.3 | 59.9 | 105.2 | 59.8 |
| 2021 | 225 | 8220340.4 | 5924280 | 4175168 | 28672 | 18661.5 | 5511.2 | 75.9 | 133.3 | 75.8 |
| 2022 | 225 | 9612842.1 | 6925662 | 4880897 | 33528.9 | 21815.8 | 6442.8 | 88.8 | 155.8 | 88.6 |
| 2020 | 375 | 6381193.3 | 4205502 | 3294191 | 22627.8 | 13247.3 | 4348.3 | 59.9 | 94.6 | 59.8 |
| 2021 | 375 | 8085681.1 | 5330194 | 4175168 | 28672 | 16790.1 | 5511.2 | 75.9 | 119.9 | 75.8 |
| 2022 | 375 | 9455371.8 | 6231157 | 4880897 | 33528.9 | 19628.1 | 6442.8 | 88.8 | 140.2 | 88.6 |
| 2020 | 650 | 6168647.8 | 3908037 | 3294191 | 22627.8 | 12310.3 | 4348.3 | 59.9 | 87.9 | 59.8 |
| 2021 | 650 | 7816362.3 | 4953177 | 4175168 | 28672 | 15602.5 | 5511.2 | 75.9 | 111.5 | 75.8 |
| 2022 | 650 | 9140431.3 | 5790413 | 4880897 | 33528.9 | 18239.8 | 6442.8 | 88.8 | 130.3 | 88.6 |
| 2020 | 1750 | 6062375.1 | 3653067 | 3294191 | 22627.8 | 11507.2 | 4348.3 | 59.9 | 82.2 | 59.8 |
| 2021 | 1750 | 7681703 | 4630020 | 4175168 | 28672 | 14584.6 | 5511.2 | 75.9 | 104.2 | 75.8 |
| 2022 | 1750 | 8982961 | 5412633 | 4880897 | 33528.9 | 17049.8 | 6442.8 | 88.8 | 121.8 | 88.6 |
| 2020 | 7500 | 5956102.3 | 3537559 | 3294191 | 22627.8 | 11143.3 | 4348.3 | 59.9 | 79.6 | 59.8 |
| 2021 | 7500 | 7547043.6 | 4483620 | 4175168 | 28672 | 14123.4 | 5511.2 | 75.9 | 100.9 | 75.8 |
| 2022 | 7500 | 8825490.7 | 5241487 | 4880897 | 33528.9 | 16510.7 | 6442.8 | 88.8 | 117.9 | 88.6 |

| Year | Curvature | VOC truck | VOC Rail D | VOC Rail E | NO2 truck | NO2 Rail D | NO2 Rail E | PM truck | PM Rail D | PM Rail E |
| --- | --- | --- | --- | --- | --- | --- | --- | --- | --- | --- |
| 2020 | 75 | 26.4 | 98.3 | 24.5 | 303.1 | 350.9 | 119.6 | 14.7 | 24.6 | 7.6 |
| 2021 | 75 | 33.5 | 124.5 | 31 | 384.1 | 444.7 | 151.6 | 18.6 | 31.1 | 9.6 |
| 2022 | 75 | 39.2 | 145.6 | 36.2 | 449.2 | 519.9 | 177.2 | 21.7 | 36.4 | 11.3 |
| 2020 | 125 | 26.4 | 78.6 | 24.5 | 303.1 | 280.6 | 119.6 | 14.7 | 19.6 | 7.6 |
| 2021 | 125 | 33.5 | 99.6 | 31 | 384.1 | 355.6 | 151.6 | 18.6 | 24.9 | 9.6 |
| 2022 | 125 | 39.2 | 116.4 | 36.2 | 449.2 | 415.7 | 177.2 | 21.7 | 29.1 | 11.3 |
| 2020 | 225 | 26.4 | 65.4 | 24.5 | 303.1 | 233.7 | 119.6 | 14.7 | 16.4 | 7.6 |
| 2021 | 225 | 33.5 | 82.9 | 31 | 384.1 | 296.2 | 151.6 | 18.6 | 20.7 | 9.6 |
| 2022 | 225 | 39.2 | 97 | 36.2 | 449.2 | 346.3 | 177.2 | 21.7 | 24.2 | 11.3 |
| 2020 | 375 | 26.4 | 58.9 | 24.5 | 303.1 | 210.3 | 119.6 | 14.7 | 14.7 | 7.6 |
| 2021 | 375 | 33.5 | 74.6 | 31 | 384.1 | 266.5 | 151.6 | 18.6 | 18.7 | 9.6 |
| 2022 | 375 | 39.2 | 87.2 | 36.2 | 449.2 | 311.6 | 177.2 | 21.7 | 21.8 | 11.3 |
| 2020 | 650 | 26.4 | 54.7 | 24.5 | 303.1 | 195.4 | 119.6 | 14.7 | 13.7 | 7.6 |
| 2021 | 650 | 33.5 | 69.3 | 31 | 384.1 | 247.7 | 151.6 | 18.6 | 17.3 | 9.6 |
| 2022 | 650 | 39.2 | 81.1 | 36.2 | 449.2 | 289.5 | 177.2 | 21.7 | 20.3 | 11.3 |
| 2020 | 1750 | 26.4 | 51.1 | 24.5 | 303.1 | 182.7 | 119.6 | 14.7 | 12.8 | 7.6 |
| 2021 | 1750 | 33.5 | 64.8 | 31 | 384.1 | 231.5 | 151.6 | 18.6 | 16.2 | 9.6 |
| 2022 | 1750 | 39.2 | 75.8 | 36.2 | 449.2 | 270.6 | 177.2 | 21.7 | 18.9 | 11.3 |
| 2020 | 7500 | 26.4 | 49.5 | 24.5 | 303.1 | 176.9 | 119.6 | 14.7 | 12.4 | 7.6 |
| 2021 | 7500 | 33.5 | 62.8 | 31 | 384.1 | 224.2 | 151.6 | 18.6 | 15.7 | 9.6 |
| 2022 | 7500 | 39.2 | 73.4 | 36.2 | 449.2 | 262.1 | 177.2 | 21.7 | 18.4 | 11.3 |

**Raw Data Totals**

| Pollutant | year | Truck | Rail | Rail Electric |
| --- | --- | --- | --- | --- |
| CO2 | 2020 | 22627.84 | 12419.58 | 4348.332204 |
|  | 2021 | 28671.99 | 15741 | 5511.22191 |
|  | 2022 | 33528.94 | 18401.7 | 6442.784093 |
| CO | 2020 | 59.929 | 88.71132 | 59.78956781 |
|  | 2021 | 75.9367 | 112.4357 | 75.77930126 |
|  | 2022 | 88.80015 | 131.4407 | 88.58828128 |
| VOC | 2020 | 26.42076 | 55.19815 | 24.45936865 |
|  | 2021 | 33.47804 | 69.95999 | 31.00062324 |
|  | 2022 | 39.14912 | 81.78533 | 36.24066052 |
| NOx | 2020 | 303.1309 | 197.1363 | 119.5791356 |
|  | 2021 | 384.1006 | 249.8571 | 151.5586025 |
|  | 2022 | 449.1661 | 292.0905 | 177.1765626 |
| PM | 2020 | 14.65105 | 13.79954 | 7.609581358 |
|  | 2021 | 18.5645 | 17.49 | 9.644638342 |
|  | 2022 | 21.70928 | 20.44633 | 11.27487216 |
